# Effect of Trp53 gene deficiency on brain injury after neonatal hypoxia-ischemia

**DOI:** 10.18632/oncotarget.14518

**Published:** 2017-01-05

**Authors:** Ana A Baburamani, Kristina S Sobotka, Regina Vontell, Carina Mallard, Veena G Supramaniam, Claire Thornton, Henrik Hagberg

**Affiliations:** ^1^ Perinatal Brain Injury Group, Centre for the Developing Brain, Division of Imaging Sciences and Biomedical Engineering, King's College London, King's Health Partners, St. Thomas’ Hospital, London, United Kingdom; ^2^ Perinatal Center, Institute of Neuroscience and Physiology, Clinical Sciences, Sahlgrenska Academy, University of Gothenburg, Sweden

**Keywords:** p53, hypoxia-ischemia, mitochondria, cell death, brain injury

## Abstract

Hypoxia-ischemia (HI) can result in permanent life-long injuries such as motor and cognitive deficits. In response to cellular stressors such as hypoxia, tumor suppressor protein p53 is activated, potently initiating apoptosis and promoting Bax-dependent mitochondrial outer membrane permeabilization. The aim of this study was to investigate the effect of *Trp53* genetic inhibition on injury development in the immature brain following HI. HI (50 min or 60 min) was induced at postnatal day 9 (PND9) in *Trp53* heterozygote (het) and wild type (WT) mice. Utilizing Cre-LoxP technology, CaMK2α-Cre mice were bred with *Trp53*-Lox mice, resulting in knockdown of *Trp53* in CaMK2α neurons. HI was induced at PND12 (50 min) and PND28 (40 min). Extent of brain injury was assessed 7 days following HI. Following 50 min HI at PND9, *Trp53* het mice showed protection in the posterior hippocampus and thalamus. No difference was seen between WT or *Trp53* het mice following a severe, 60 min HI. Cre-Lox mice that were subjected to HI at PND12 showed no difference in injury, however we determined that neuronal specific CaMK2α-Cre recombinase activity was strongly expressed by PND28. Concomitantly, *Trp53* was reduced at 6 weeks of age in KO-Lox *Trp53* mice. Cre-Lox mice subjected to HI at PND28 showed no significant difference in brain injury. These data suggest that p53 has a limited contribution to the development of injury in the immature/juvenile brain following HI. Further studies are required to determine the effect of p53 on downstream targets.

## INTRODUCTION

Hypoxic-ischemic encephalopathy (HIE), caused by a lack of oxygen or blood flow to the brain around the time of birth, affects 1-2 in every 1000 live births in the UK [1] and far more in the developing world [2]. The consequences for babies and parents affected by HIE are devastating with a high proportion of survivors suffering from severe and long-lasting motor and cognitive impairments [3]. Neuroprotective therapy with hypothermia improves outcome and prevents death or neurological outcome in one in seven babies which represents a breakthrough in the care of these vulnerable neonates [4]. Although therapeutic hypothermia is not universally successful, it provides proof-of-concept that intervention following injury can offer significant benefit. However, in order to provide additional more effective therapies we urgently require a more thorough understanding of the underlying pathophysiology.

Brain injury develops in different phases following hypoxia-ischemia (HI) depending on the brain region and severity of the primary insult [5, 6]. Cell death after neonatal brain injury is characterized morphologically by a mixed necrotic, necroptotic, autophagic and apoptotic phenotype [7–10], with predominantly neurons vulnerable to insult in the near term brain. However, data from our lab and others strongly suggest that the common thread linking these diverse mechanisms is mitochondrial perturbation [11–13].

HI triggers multiple signaling events such as excitotoxicity, release of reactive oxygen species and increase in intracellular calcium [5, 6, 11, 12]. Bcl-2 family member Bax is activated and translocates to the mitochondria where it and family member Bak homo-oligomerise forming pores ultimately leading to mitochondrial outer membrane permeabilization (MOMP) [5]. MOMP allows the efflux of pro-apoptotic proteins such as cytochrome c and apoptosis inducing factor (AIF) into the cytosol [13–17] thereby initiating a cascade resulting in activation of caspases, degradation of DNA and ultimately cell death [14, 15, 18].

It is, however, still unclear how MOMP is initiated. Besides changes in pro- vs. anti-apoptotic Bcl-2 protein ratio, several other upstream regulators may be involved such as caspase-2 and c-Jun N-terminal kinase (JNK) [19, 20]. The tumor suppressor protein p53 (*Trp53* mouse gene) is one such candidate recently implicated in mediating neuronal cell death observed after neonatal HI. Nuclear p53 triggers apoptosis via multiple pathways, for example cell cycle arrest, the regulation of autophagy [21], through transactivating pro-apoptotic and repressing anti-apoptotic genes [22]. In addition, p53 also has cytoplasmic actions at the mitochondrial level and can promote Bax-dependent MOMP [23, 24]. Nuclear export of p53 depends on mono-ubiquitination by the E3 ubiquitin-ligase Mdm2 and once at the mitochondria, p53 is deubiquitinated by the ubiquitin specific peptidase 7 (HAUSP) [25]. Cytosolic p53 is probably inactivated through binding to Bcl-XL and/or Mcl-1 [24, 26]. P53 is, however, liberated from Bcl-XL/Mcl-1 by high levels of p53 up-regulated modulator of apoptosis (PUMA) and can subsequently interact with Bax at the outer mitochondrial membrane.

There are several reasons to suspect that p53 is important in the context of Bax-dependent MOMP and cell death in the neonatal brain. Firstly, the expression of p53 and several of its downstream genes are induced in the brain after neonatal HI [27, 28]. Secondly, p53, PUMA and Noxa accumulate in the cytosol and p53 translocates to mitochondria after neonatal HI [28–30]. Thirdly, inhibiting NFκB (a regulator of p53) by blocking IκB-kinase with a Nemo binding domain TAT peptide reduces p53 levels and subsequently cytochrome c release and brain injury in a neonatal HI mouse model [29–31]. Finally, Pifithrin-μ (PFT-μ, inhibitor of the mitochondrial actions of p53) [32] blocks mitochondrial accumulation of p53, cytochrome c release and subsequent activation of caspase-3, substantially reducing neonatal brain injury [31]. However, PFT-μ also has effects on microglial activation [33] and heat shock proteins (HSP) [34] and we still lack convincing genetic proof that p53 is involved.

The aim of the present study is to apply a two-pronged approach to evaluate whether, firstly, a partial gene deficiency of *Trp53*, comparing *Trp53* heterozygote mice with wild types, confer neuroprotection in a neonatal model of term HI and, secondly, conditional neuronal deletion of *Trp53* (utilizing CaMK2αCre – Lox *Trp53* mice [35]) affects the extent of HI brain injury in neonatal and juvenile mice.

## RESULTS

### *Trp53* hets are less injured at posterior levels of the brain following moderate (50 min) but not severe (60 min) of hypoxia-ischemia at postnatal day (PND) 9

At PND 9, mixed genotype litters of both sexes were randomly subjected to either a moderate 50 min or severe 60 min episode of HI. Loss of grey matter (MAP-2 staining) and white matter (MBP staining) was evaluated 7 days following HI.

**Figure 1:**
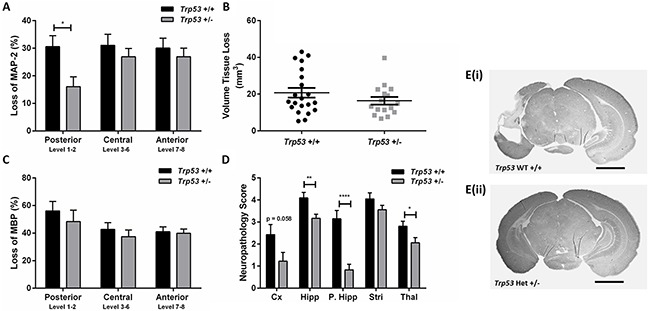
Brain injury assessment of *Trp53* WT (+/+) and Het (+/−) mice 7 days following 50 min HI at PND 9 MAP-2 tissue loss **A**. volume tissue loss **B**. MBP loss **C**. and neuropathology scores **D**. were assessed at 8 levels of the brain in *Trp53* WT (black bars; n=21) and *Trp53* het (grey bars; n=16). MAP-2 stained images at posterior levels of the brain from *Trp53* WT +/+ **E(i)**. and *Trp53* het +/− **E(ii)**. Mean ± SEM. * p< 0.05, ** p < 0.01, **** p<0.0001. Cortex (Cx), Hippocampus (Hipp), posterior hippocampus (P. Hipp), Striatum (Stri), Thalamus (Thal). Scale bar represents 2 mm.

Following moderate (50 min) HI, no differences in body weight were observed between genotypes at PND 9 and PND 16. Analysis of the infarct revealed that *Trp53* hets (n=18; females n=8, males n=10) showed significantly less MAP-2 tissue loss in the posterior levels of the brain (Figure 1A, E) compared with *Trp53* WT (n=21; females n=8, males n=13; 16.1 ± 3.5% *vs* 30.6 ± 4.0%, p<0.05). No differences were observed at the central or anterior levels of the brain. No differences were observed in volume tissue loss (Figure 1B) or loss of MBP staining (Figure 1C).

The atrophy and infarction (0 to 6) for each region of interest (cortex, hippocampus, striatum and thalamus) were assessed to obtain a neuropathology score of the left, injured, hemisphere (Figure 1D) [36]. *Trp53* hets showed significantly less injury particularly in the posterior region of the hippocampus and the thalamus. A trend towards less injury was also seen in the cortex (p=0.058). No differences were observed in the striatum. In spite of the quite marked protection in the *Trp53* het mice in the posterior part of the brain, we observed no difference in overall volume tissue loss as the central and anterior part of the brain including striatum and most parts of cerebral cortex were unaffected by p53 genotype.

**Figure 2:**
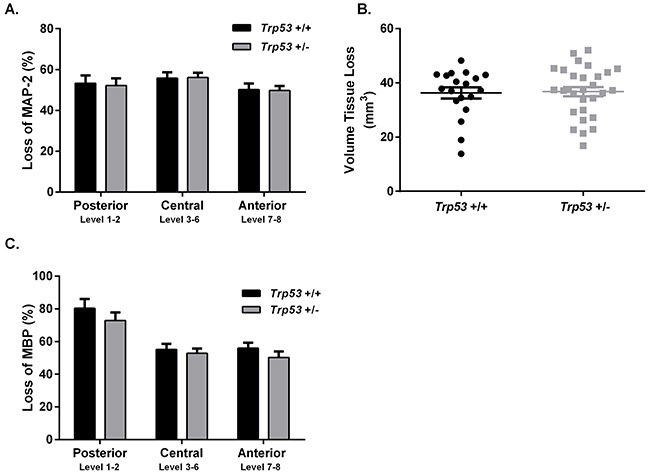
Brain injury assessment of *Trp53* WT (+/+) and Het (+/−) mice 7 days following 60 min HI at P9 MAP-2 tissue loss **A**. volume tissue loss **B**. and MBP loss **C**. were assessed at 8 levels of the brain in *Trp53* WT (black bars; n=20) and *Trp53* het (grey bars; n=29). Mean ± SEM.

Following a severe (60 min) duration of HI there were no differences between *Trp53* WT (n=20; females n=11, males n=9) and *Trp53* hets (n=29; females n=15, males n=14) when assessed at PND 16 for MAP-2 tissue loss (Figure 2A), volume tissue loss (Figure 2B) or MBP loss (Figure 2C).

### No effect of conditional knock-down of *Trp53* on injury at PND 12

In order to reduce any influence on phenotype due to the global absence of p53, we generated a conditional *Trp53* knockout mouse in which deletion of *Trp53* was limited to neurons in the forebrain. We chose to combine *Trp53*-lox animals with mice expressing Cre recombinase under the control of the calcium/calmodulin-dependent protein kinase II alpha (CaMK2α) promoter. The CaMK2α promoter is reported to facilitate early, neuronal expression of the gene of interest [37]. PND12 mixed litters consisting of WT Lox *Trp53*^+/+^ (n=16; females n=7, males n=9) and KO Lox *Trp53*^f/+^ (n=24; males n=13, females n=11) were subjected to 50 min of HI. There was, however, no difference between genotypes in MAP-2 tissue loss overall or in the hippocampus (data not shown).

### CaMK2α Cre activation is initially detected at PND 19

**Figure 3:**
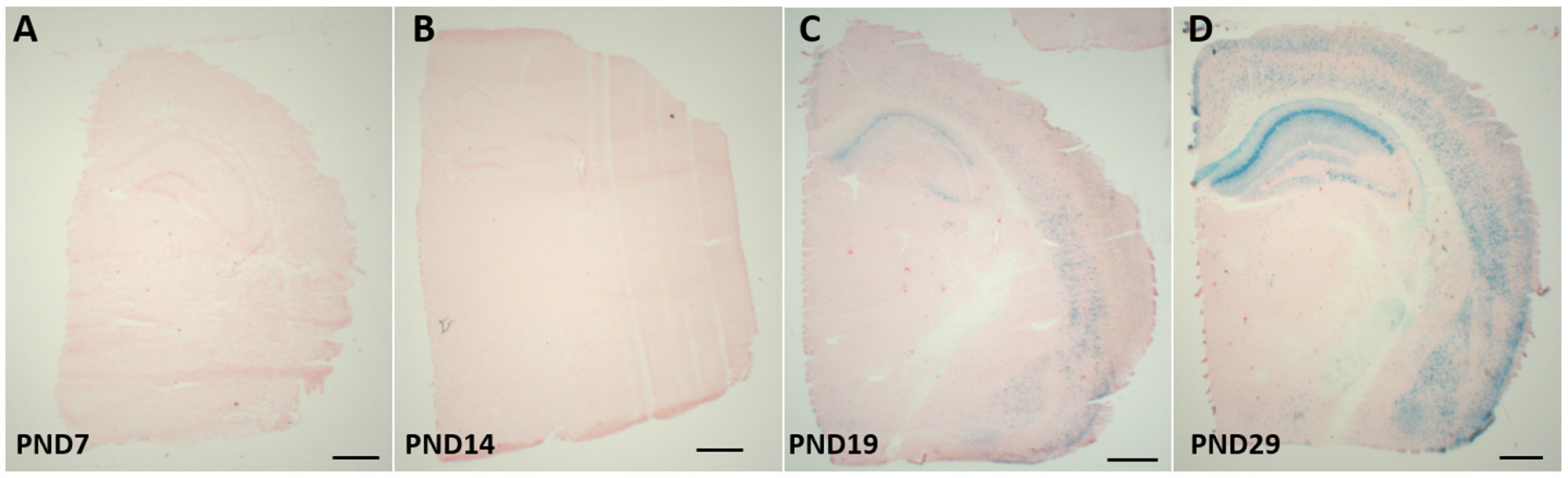
β-gal expression in the brains of CaMK2α Cre - Rosa26 reporter mice CaMK2α Cre mice were bred with ROSA26 reporter mice; brains were collected at postnatal day (PND) 7 **A**. PND 14 **B**. PND 19 **C**. and PND29 **D**. B-gal staining is representative of cre-recombinase activity, which occurs from PND 19. Scale bar represents 1000 μm.

In order to investigate the regional and age-dependent expression of Cre recombinase activity which may have influenced the previous result, CaMK2α-Cre mice were bred with Rosa26 LacZ reporter mice. X-gal staining of β-galactosidase revealed no positive Cre recombinase activity (shown by blue stained cells) at PND 7 or PND 14. At PND 19, blue stained cells were present in the CA1 and CA2 regions of the hippocampus, with subtle staining seen in the cortex. By PND 28 strong staining was present throughout the hippocampus and cortex, at the level of approximately Bregma -2.12mm. This was consistent in pups generated from paternal (bred with a ROSA26 female) or maternal (bred with a ROSA26 male) Cre inheritance (Figure 3).

### *Trp53* messenger RNA expression in CaMK2α Cre-*Trp53* Lox mice

**Figure 4:**
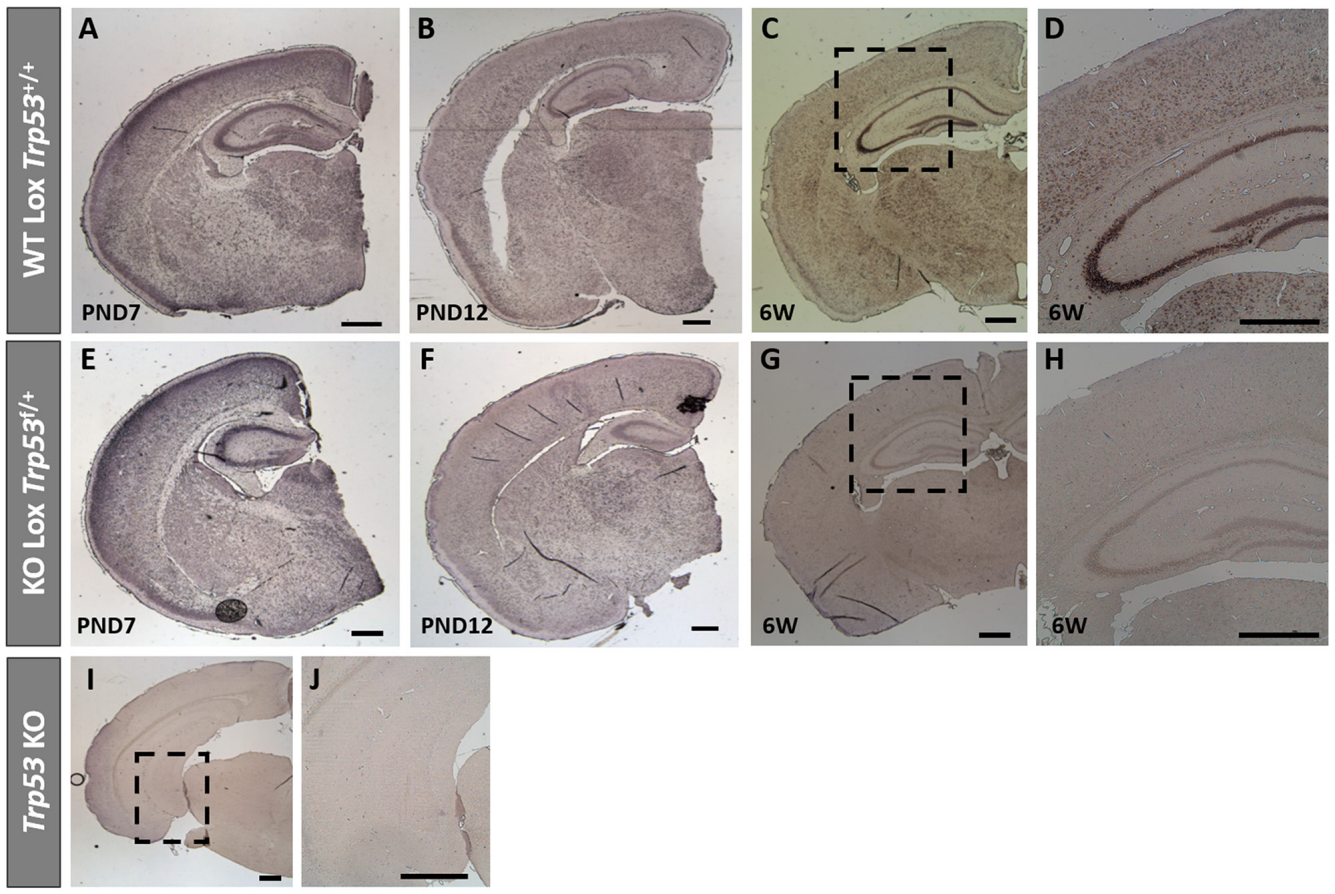
*Trp53* *in situ* hybridization on brains from WT Lox *Trp53*^+/+^, KO Lox *Trp53*^f/+^ and *Trp53* KO *In situ* hybridization of *Trp53* in WT Lox *Trp53*^+/+^ **A-D**. KO Lox *Trp53*^f/+^ **E-H**. and traditional *Trp53* KO **I, J**. at postnatal day (PND) 7 **A, E**. PND 12 **B, F**. 6 weeks of age **C, G**. and [higher magnification **D, H**.]. Traditional *Trp53* KO **I**. higher magnification **J**. shows no *Trp53* staining. Scale bars represent 500 μm.

As antibodies directed towards mouse p53 are unreliable for immunohistochemistry (data not shown), to confirm CaMK2α Cre-mediated deletion of *Trp53* from neurons, *in situ* hybridization was conducted for *Trp53* mRNA in WT Lox *Trp53*^+/+^ and KO Lox *Trp53*^f/+^ (Figure 4). Brains were assessed at PND 7, PND 12 and at 6 weeks of age. *Trp53* mRNA expression was present at PND 7 and PND 12 in both genotypes (Figure 4A, 4B, 4E, 4F). At 6 weeks of age WT Lox *Trp53*^+/+^ (Figure 4C, 4D) exhibited *Trp53* mRNA staining in the hippocampus, in contrast to KO Lox *Trp53*^f/+^ that showed sparse hippocampal *Trp53* expression (Figure 4G, 4H). This staining was consistent with the regions where we observed Cre-recombinase activation, shown by x-gal staining (Figure 3D). Traditional *Trp53* KO mice served as a negative control and no *Trp53* mRNA staining was observed throughout the brain (Figure 4I, 4J).

### Conditional knock-down of *Trp53* was not protective against hypoxic-ischemic injury at PND 28

**Figure 5:**
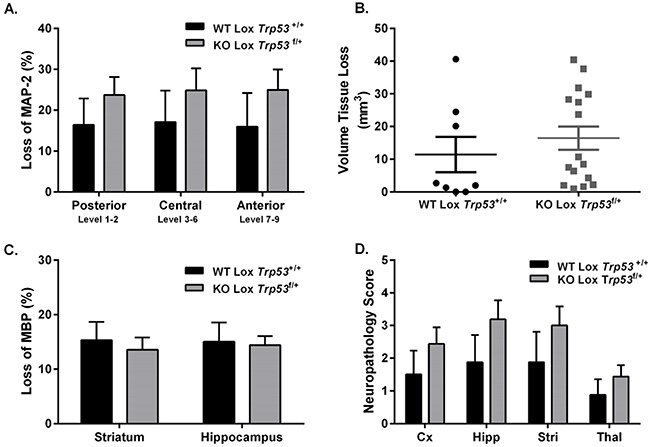
Brain injury assessment of WT Lox *Trp53*^+/+^ and KO Lox *Trp53*^f/+^ mice 7 days following 40 min HI at PND 28 MAP-2 tissue loss **A**. volume tissue loss **B**. MBP loss **C**. and neuropathology score **D**. were assessed in WT Lox *Trp53*^+/+^ (black bars; n=8) and KO Lox *Trp53*^f/+^ (grey bars; n=16). Mean ± SEM. Cortex (Cx), Hippocampus (Hipp), Striatum (Stri), Thalamus (Thal).

Based on the LacZ reporter staining and the *in situ* data showing that *Trp53* expression was significantly reduced at PND 28, we induced 40 min of HI at PND 28 in mixed litters from Cre-Lox mice. No differences in brain injury were seen between WT Lox *Trp53^+/+^* (n=8; females n=4, males n=4) and KO Lox *Trp53^f/+^* (n=16; females n=8, males n=8) 7 days following HI. No differences were observed in loss of MAP-2 (total group or separated for sex), volume tissue loss or loss of MBP (Figure 5). We also performed neuropathology scoring of the different regions and found that brain injury in the juvenile brain (PND 28) was observed in the same regions (hippocampus, thalamus, striatum and cortex) as in the immature (PND 9 or PND 12) brain which agree with previous work [38]. However, we found no difference between genotypes following HI at PND 28.

## DISCUSSION

In summary, we found that heterozygote *Trp53* gene deficiency conferred partial neuroprotection in the posterior part of the brain including the posterior part of the hippocampus, cerebral cortex and thalamus in response to a moderate insult of 50 min of HI but not following a more severe 60 min insult of HI in PND 9 mice. Brain injury was not affected in neonatal (PND 12) or juvenile (PND 28) mice by a conditional neuron-selective *Trp53* gene deletion.

The moderate neuroprotection seen in the posterior part of the brain in neonatal *Trp53* het mice contrasts with the profound tissue protection seen in adult mice. Adult *Trp53* het mice develop ~80% smaller brain injury compared with WT controls after ischemia [39]. There are several possible explanations for the developmental difference in vulnerability dependent on p53. It was recently discovered that in the adult brain p53 interacts with cyclophilin D (CyD) and the complex induces opening of the mitochondrial permeability transition (MPT) pore leading to necrotic cell death [39]. Hence downregulation of p53 or blockage of CyD with cyclosporin A reduces ischemic injury in adults [40, 41]. However, in the setting of the immature brain the situation is different. Ischemic necrosis doesn't seem to depend on CyD or MPT as cyclosporin A or *CyD* gene deficiency do not confer neuroprotection [14, 42]. Instead, RIP kinase-dependent necrosis is proposed to be critical for this mode of cell death [43]. Speculatively, partial p53 gene depletion is markedly protective in the adult brain as CyD dependent necrosis and apoptosis are both attenuated. In the immature brain, Bax-dependent apoptotic cell death is reduced in *Trp53* het mice reducing vulnerability but the degree of protection is limited as necrosis is unaffected. It also could explain why p53 genotype did not influence brain injury after a severe insult of 60 min HI in the neonates, as necrosis becomes the predominant mode of cell death even in the immature brain under those conditions [7].

Whilst the ideal comparison to access the effect of genetic knockdown would be between *Trp53* KO and WT pups, it is widely reported that traditional *Trp53* KO mice develop malignancy throughout the body from a young age [44]. Also, whilst all our breeding paradigms were generating mixed genotype litters, only very few *Trp53* KO (n=6) pups were born. These pups were subjected to 60 min HI and no difference between genotypes in injury was seen (data not shown). We therefore compared *Trp53* het with *Trp53* WT, which, in two studies in the adult mouse brain have shown that a partial knockdown of p53 is sufficient to protect the brain following focal ischemia [39, 45].

In order to reduce secondary phenotypic influences in conventional knock-out mice we also adopted the Cre-LoxP system to allow for conditional gene deletion in predominantly hippocampal and cortical neurons, regions that are vulnerable to near term hypoxic-ischemic injury. We chose the promoter CaMK2α [35] where the mRNA for CaMK2α is expressed in most brain regions at postnatal day 4-14 [46]. This significant increase in expression from second to third postnatal week is thought to correlate with active forebrain synaptogenesis [47]. However, according to our assessment of cre-recombinase activity, through breeding with a ROSA26 reporter mouse and staining for X-gal, CaMK2α cre-recombinase activation was only present from PND 19, consistent with Tsien et al., [37]. In addition, our *in situ* hybridization results also showed *Trp53* expression was apparent until PND 12, which could explain the lack of neuroprotection in Cre-LoxP mice at PND 12. But it was surprising that HI brain injury was unaffected by conditional neuronal deletion of *Trp53* also at PND 28 considering the marked cre-recombinase activation and loss of *Trp53* gene expression at this age and the protective effect of conventional gene deletion in adults [39, 45].

This lack of effect suggests that selective gene deletion in neurons is insufficient to alter the extent of pan-cellular focal lesions produced in this HI model in mice at PND 9-60 [38, 48] and indeed, increasing evidence implies that p53 impacts glial function in development and neurodegeneration [49]. Enhanced expression of p53 in astrocytes has been reported 7 days following adult cerebral ischemia in rodents [50] and nitric oxide-induced cell death in astrocytes is mediated via p53 [51]. Overexpression of p53 has also been implicated in oligodendrocyte cell death *in vitro* and p53 expression is strongly associated with oligodendrocyte destruction in lesions of adult patients with multiple sclerosis [52, 53]. In addition to an essential role in cell death, p53 is also associated with oligodendrocyte precursor cell (OPC) development, where the optic nerve in *Trp53* knockout mice at PND 7 showed an increase in OPC number but a decrease in the number of oligodendrocytes [54].

In models of demyelination, genetic knock down (*Trp53* knockout mice) or pharmacological inhibition (PFT-α) of p53 resulted in preservation of myelinated fibres and increased oligodendrocyte survival [55]. This study also found decreased microglial activation and recruitment to the site of injury (corpus callosum) [55]. P53 expression in microglia has been shown to promote a pro-inflammatory phenotype that is damaging to neuronal synapses [56]. Numerous studies have also found that reduced expression of p53, such as in *Trp53* knockout mice or following inhibition (PFT-μ), results in increased expression of anti-inflammatory genes and functions leading to tissue repair and phagocytosis, suggesting that in microglia, p53 modulates the activation state [33, 55, 57]. These studies indicate that the role of p53 in other cell types can significantly contribute to the progression of injury, and could explain the lack of protection following neuronal knockdown of *Trp53*. Additionally, this could have a greater impact in the adult and ageing brain.

Our findings strongly suggest that the CaM2Kα, from the T29-1 line, is not the ideal choice for neonatal experiments considering Cre, under the promoter of CamK2α, is not active till after PND 14. Whilst these experiments were being conducted the online cre repository was being characterized as a global resource [58]. As a consequence, it was recognized that Cre inherited from the maternal or paternal line can differ in expression, although we did not see this. In addition, it is important to recognize that Cre expression can differ between litter mates and other organs may be affected, all of which are important considerations when choosing a Cre line.

The partial neuroprotection we found in *Trp53* het mice in response to moderate HI (50 min HI) is more subtle and limited to the posterior part of the brain in response to moderate degrees of HI when compared with our previous demonstration that PFT-μ reduces brain injury globally after neonatal HI [31]. The degree of neuroprotection exerted by PFT-μ was considerably more pronounced than by heterozygote *Trp53* gene deficiency. This discrepancy could be due to that the degree of gene deletion was only partial (comparing *Trp53* het vs. *Trp53* WT) and that the cerebroprotective effect of PFT-μ is complex and not solely explained by its blockage of p53 interaction at the outer mitochondrial membrane. PFT-μ interacts with HSPs [34] and modulates the immuno-inflammatory response [33] which certainly could contribute to its neuroprotective efficacy in HI. In addition to PFT-μ, PFT-α has been used to inhibit the transcriptional activity of p53 which results in decreasing p53 DNA binding activity [59, 60]. Treatment with PFT-α given prior to [61], 1-6h following [62], or 6–9 days [63] following adult cerebral ischemia resulted in neuroprotection.

In summary, we have found that partial knockdown of *Trp53* is protective following a moderate hypoxic-ischemic insult in posterior regions of the immature brain. More severe insults and conditional neuronal knockdown in the juvenile brain did not show any level of protection. Whilst p53 is a key mediator of neuronal cell death, acting upstream of Bax-dependent MOMP in the neonatal brain, further studies evaluating mediators of p53 activation could reveal new therapeutic targets.

## MATERIALS AND METHODS

### Ethics statement

All animal experimentation conducted in accordance with Department of Agriculture (Jordbruksverket, Sweden) and approved by the Gothenburg animal ethics committee (Application 52-2012).

### Animals

Mice were bred in-house, kept in a 12 hour light/dark cycle, and fed standard laboratory chow diet and drinking water, *ad libitum* (Experimental Biomedicine, University of Gothenburg, Sweden). All mice were purchased from The Jackson Laboratory.

### Traditional Trp53 Knockouts

*Trp53* heterozygote (Het; +/−) mice (Stock #002101; B6.129S2-*Trp53^tm1Tyj^*/J) [44] were bred together or with wild types (WT; +/+ [generated from *Trp53* het-het cages]) to produce mixed genotypes; *Trp53* knockout (KO; -/−), *Trp53* het and *Trp53* WT. *Trp53* knockout mice tend to develop tumors in early life and were therefore not used in these studies. Mixed litters (*Trp53* het and *Trp53* WT) were used for HI experiments as previously described in adult experiments on ischemia [39].

### Cre-LoxP conditional knockouts

Neuronal specific knockdown of *Trp53* was achieved utilizing Cre-LoxP technology. Briefly, LoxP mice containing the (floxed) *Trp53* allele (Lox; Cre^-/−^ Lox^+/+^, strain #008462; B6.129P2-*Trp53^tm1Brn^*/J) were bred with mice expressing Cre recombinase under the promoter of calcium/calmodulin-dependent protein kinase II alpha, of the T29-1 line (Cre; Cre^+/+^ Lox^-/−^, CaMK2α-Cre T29-1, Stock# 005359; B6.Cg-Tg(CaMK2α-cre)T29-1Stl/J). First generation (Cre^+/+^ Lox^-/−^ x Cre^-/−^ Lox^+/+^) breeding resulted in heterozygote Cre-Lox mice (Cre^+/−^ Lox^+/−^). These were subsequently bred with Lox mice to produce a second generation of mixed genotypes [Het Cre-Lox (Cre^+/−^ Lox^+/−^), Hom Lox (Cre^-/−^ Lox^+/+^), Het Lox (Cre^-/−^ Lox^+/−^) and neuronal specific KO of *Trp53* (KO Lox *Trp53*^f/+^; Cre^+/−^ Lox^+/+^). These KO Lox *Trp53*^f/+^ were bred with Lox mice to generate mixed litters: WT Lox *Trp53*^+/+^ (Cre^-/−^ Lox^+/+^) and KO Lox *Trp53*^f/+^ (Cre^+/−^ Lox^+/+^) that were used for HI experiments.

### Cre-ROSA26 characterization

Currently the online cre portal (http://www.creportal.org, http://www.informatics.jax.org/allele/MGI:2177650) only contains information regarding cre recombinase activity at ages Embryonic (E) 10.5, E15.5, PND 7 and PND 56. To further characterize CaMK2α-cre T29-1 cre activity we bred with ROSA26 LacZ reporter mice (Rosa26^+/+^; strain #003474 (B6.129S4-*Gt(ROSA)26Sor^tm1Sor^*/J) [58], which generated Cre^+/−^ Rosa26^+/−^. Both male and female Cre mice were used to breed with Rosa26 reporter mice to further assess whether maternal or paternal cre inheritance was different [58]. At PND 7, PND 14, PND 19 and PND 29 brains were harvested and frozen at -80°C. Coronal sections were cut on a cryostat at 10 μm. β-galactosidase staining was done following manufacturer instructions (Cat number: K1465-01, Invitrogen, Sweden). Sections were visualized on a Zeiss Microscope (Model Axio Imager 2, Zeiss, Germany) and captured on an AxioCam MRm Camera (Zeiss, Germany) using Zen Software (Zeiss, Germany).

### Genotyping

The genotype of WT, Het and KO mice were determined by polymerase chain reaction (PCR) of genomic DNA extracted from tail clips by RedExtract-N-AMP Tissue PCR Kit (Sigma-Aldrich, USA). All primers were ordered from Eurofins MWG GmbH (Ebersberg, Germany). For traditional *Trp53* transgenic mice; each PCR reaction (20μl) contained 2 μl (<250 ng DNA) and was made following KAPA Taq DNA Polymerase manufacturer's instructions (KAPA BioSystems, USA) and primer concentrations of 0.08 μM IMR7777, 1μM IMR7778, 1.17 μM IMR8306. For Cre-Lox and Rosa26 genotyping: each PCR reaction (10 μl) contained 1 μl (50 – 100 ng) of genomic DNA, 5 μl of REDExtract-N-Amp PCR Reaction mix and all primers were used at a concentration of 1 μM. The PCR products were separated on a 1.5% agarose/0.5 x Tris-borate-ethylenediaminetetraacetic acid gel containing SYBR® Safe DNA gel stain (Invitrogen, Life Technologies Sweden). Primer details, PCR programs and expected results are listed in Table 1.

**Table 1:** Primers and PCR programs used for genotyping transgenic mice

Gene			Sequence 5’ → 3’	Concentration (μM)	PCR Program	Result
P53	IMR7777	WT Forward	ACA GCG TGG TGG TAC CTT AT	0.08	94°C for 3 min 35 cycles: [94°C for 30 sec; 66°C for 60 sec; 72°C for 90 sec] 72°C for 2 minutes.	WT: 450 bp Het: 450bp and 650bp Mutant: 650 bp
	IMR8306	Mutant Forward	CTA TCA GGA CAT AGC GTT GG	1.17		
	IMR7778	Common	TAT ACT CAG AGC CGG CCT	1		
Cre	IMR1084	Transgene	GCG GTC TGG CAG TAA AAA CTA TC	1	94°C for 5 min 35 cycles: [94°C for 30 sec; 52°C for 60 sec; 72°C for 60 sec] 72°C for 2 min	Transgene: ~100bp Internal positive control: 324 bp
	IMR1085	Transgene	GTG AAA CAG CAT TGC TGT CAC TT	1		
	IMR7338	Internal positive control Forward	CTA GGC CAC AGA ATT GAA AGA TCT	1		
	IMR7339	Internal positive control Reverse	GTA GGT GGA AAT TCT AGC ATC ATC C	1		
P53 Lox	IMR8543	Forward	GGT TAA ACC CAG CTT GAC CA	1	94°C for 5 min 35 cycles: [94°C for 30 sec; 56°C for 60 sec; 72°C for 60 sec] 72°C for 5 min	WT: 270 bp Het: 270 bp and 390 bp Mutant: 390 bp
	IMR8544	Reverse	GGA GGC AGA GAC AGT TGG AG	1		
Rosa26	IMR8545	WT Reverse	GGA GCG GGA GAA ATG GAT ATG	1	94°C for 3 min 35 cycles: [94°C for 30 sec; 65°C for 60 sec; 72°C for 60 sec] 72°C for 2 min	WT: ~650 bp Het: 340 bp and ~650 bp Mutant: 340 bp
	IMR8052	Mutant Reverse	GCG AAG AGT TTG TCC TCA ACC	1		
	IMR8545	Common	AAA GTC GCT CTG AGT TGT TAT	1		

### *In situ* hybridization

Mouse *Trp53* complementary DNA was cloned from mouse brain total RNA by reverse transcription–polymerase chain reaction (Multiscribe; Applied Biosystems), as recommended by the manufacturer. Second-round amplification was performed with REDTaq (Sigma-Aldrich, Dorset, United Kingdom) and 0.5 μmol/L forward (5′-GGC-AAC-TAT-GGC-TTC-CAC-C-3′) and reverse (5′-CTC-CGT-CAT-GTG-CTG-TGA-C-3′) primers (based on accession number NM_001127233) using the following cycling parameters: 95°C for 5 minutes, 1 cycle; 95°C for 30 seconds; 65°C for 30 seconds; 72°C for 1 minute, 30 cycles; 72°C for 5 minutes. Each complementary DNA product was directionally cloned into TOPO II plasmid (Life Technologies, Carlsbad, CA), which incorporates T7 and SP6 RNA polymerase promoters flanking the cloning region defined by the *Hin*dIII or *Xba* 1 restriction enzyme site. Plasmids were sequenced, linearized, and transcribed with T7 (antisense) or SP6 (sense) RNA polymerases (Sigma) to yield digoxigenin-dUTP–labelled riboprobes in accordance with the manufacturer's protocol (Ambion, Life Technologies). Transcription was performed for 24 hours at 37°C. The template complementary DNA was digested away by RNase-free DNase (2 U/μL, 15 minutes), and the riboprobes were purified (Megaclear Purification and Filtration System; Ambion, UK) and quantified by spectrophotometry and electrophoresis.

### In situ hybridization procedure

WT Lox *Trp53*^+/+^ and KO Lox T*rp53*^f/+^ pups were killed at PND 7, PND 12 and at 6 weeks of age, perfused with 0.9% physiological saline, followed by 5% buffered formalin. Brains were then paraffin embedded and 6 μm sections were cut at the level of the hippocampus. The in situ hybridization procedure was conducted as previously described [64], with overnight incubation occurring at 56°C and the 2× saline-sodium citrate washes occurring for 30 minutes at 55°C. Adjacent slides incubated with the sense probe and hybridization buffer alone were used as negative controls for specificity. Sections were visualized on a Leica Microscope (Model DM6000B, Leica Microsystems, UK) and captured on a MBF Bioscience Camera (Model Cx9000, MBF Bioscience, USA) using Stereo Investigator software (v5.65, MBF Bioscience, USA).

### Induction of hypoxia-ischemia in neonatal and juvenile mice

Traditional *Trp53* knockout mice at PND 9 and Cre-Lox mice at PND 12 and PND 28 were subjected to unilateral HI, according to the Rice–Vannucci model as previously described [36, 38, 65]. Briefly, the left common carotid artery was isolated and ligated. Pups recovered for 1 h in the parent cage. The litters were then placed in a chamber with a humidified hypoxic gas mixture (10% oxygen in nitrogen, 36°C) for PND 9 mice, 50 min (moderate hypoxia) or 60 min (severe hypoxia), PND 12 for 50 min, and PND 28 mice for 40 min. After hypoxic exposure, pups were returned to their biological dams until the conclusion of the experiment (7d post-HI). Mortality rates from the end of HI to +7 days post-HI were low: PND 9; 50 min and 60 min = 0; PND 12 for 50 min HI = 1/41 and PND 28 40 min HI = 2/28. The genotypes of these mice pups was not determined.

### Brain injury assessment

Pups were killed, tail samples were collected for genotyping and mice were perfused intracardially with 0.9% physiological saline followed by 5% buffered formaldehyde (Histolab; Histolab AB, Sweden) as previously described [14, 66]. Paraffin-embedded tissue blocks were serially cut into 7 μm coronal sections on Superfrost® plus slides (Thermo Scientific, Sweden) with every 50th section stained. Microtubule associated protein-2 (MAP-2), a neuronal marker expressed in neurons and dendrites, and myelin basic protein (MBP), a major constituent of the myelin sheath of oligodendrocytes where loss of staining indicated infracted areas and MBP associated white matter loss respectively as previously described [14, 66]. Images were taken on a Nikon Optiphot-2 (Nikon, Japan) using an AVT Dolphin camera (Model 1 F145g, Nikon Germany). Positive stained areas were measured using the Olympus Micro Image analysis software system (V4.0, Olympus Optical, Tokyo, Japan).

Loss of MAP-2 and MBP staining was determined by subtracting the MAP-2 or MBP-positive area in the ipsilateral (injured) hemisphere from the contralateral (non-injured) hemisphere, and values expressed as a percentage of tissue loss from the contralateral hemisphere. Total volume loss was calculated according to the Cavalieri Principle; V = ΣA x P x T, where V = total volume, ΣA = sum of the areas, P = the inverse of the sections sampling fraction and T = section thickness. MBP staining was measured in the subcortical white matter region, at one level of the brain (approximately at the level of Bregma -2.12 mm) [67].

Neuropathology was evaluated by an assessor blinded to genotype. A modified semi-quantitative scoring scale [36, 68] was used to assess injury in the cortex, hippocampus, posterior hippocampus, striatum and thalamus. Each region was graded for degree of atrophy 0 to 3 (0 being no atrophy) and cellular infarction and necrosis 0 to 3 (0 indicating no injury), resulting to each region receiving a score between 0 – 6.

### Data analysis

Data are expressed as mean ± SEM. All statistical analysis was performed on GraphPad Prism 6 Software (GraphPad Software, San Diego, USA), with statistical significance set at p< 0.05. Loss of MAP-2, loss of MBP, volume tissue loss and neuropathology score were assessed by a student's t-test. Loss of MAP-2, separated for sex was assessed by a One-Way ANOVA for each region. If significant, a Tukey's post-hoc analysis was conducted.
